# Alemtuzumab-Induced Resistant Thyroid Storm in a Patient With Graves’ Disease Requiring Urgent Thyroidectomy: A Case Report

**DOI:** 10.7759/cureus.97284

**Published:** 2025-11-19

**Authors:** Shahzad Akbar, Samraiz Nafees, Muhammad Haroon Riasat, Ali Javeed, Khaled Zamari, Jagannath Gopalappa, Vijay Jayagopal

**Affiliations:** 1 Endocrinology and Diabetes, Hull Royal Infirmary, Hull University Teaching Hospitals NHS Trust, Hull, GBR; 2 Internal Medicine, Scarborough General Hospital, York and Scarborough Teaching Hospitals NHS Foundation Trust, Scarborough, GBR; 3 Endocrinology and Diabetes, Scarborough General Hospital, York and Scarborough Teaching Hospitals NHS Foundation Trust, Scarborough, GBR; 4 General Practice, Gloucestershire Hospitals NHS Foundation Trust, Bath, GBR; 5 Endocrinology and Diabetes, York Hospital, York and Scarborough Teaching Hospitals NHS Foundation Trust, York, GBR

**Keywords:** alemtuzumab, graves’ disease, immune reconstitution, mutiple sclerosis, papillary thyroid carcinoma, thyroidectomy, thyroid storm, thyrotoxicosis

## Abstract

A 38-year-old woman with relapsing-remitting multiple sclerosis (RRMS) treated with alemtuzumab developed severe Graves’ thyrotoxicosis presenting with palpitations, chest tightness, dizziness, and marked tachycardia with a heart rate of 160 per minute. Despite escalating antithyroid medications and beta-blockers, her condition progressed to thyroid storm that was resistant to medical therapy. Urgent total thyroidectomy was performed after preoperative euthyroidism was achieved with Lugol’s iodine. Histopathology confirmed Graves’ disease and revealed an incidental papillary thyroid carcinoma. This case is unique because it illustrates a rare instance of alemtuzumab-induced autoimmune thyroid disease progressing to treatment-refractory thyroid storm, further complicated by an incidental papillary thyroid carcinoma, an extremely uncommon coexistence. It highlights the need for heightened vigilance in monitoring alemtuzumab-treated patients for severe thyroid dysfunction and the importance of timely surgical intervention when medical therapy fails. Overall, it emphasizes that alemtuzumab-related thyrotoxicosis can evolve into a life-threatening treatment-resistant thyroid storm, and concurrent thyroid malignancy may be found incidentally, underscoring the value of multidisciplinary management and early surgical consideration.

## Introduction

Alemtuzumab is a monoclonal antibody targeting CD52, a protein expressed on mature lymphocytes, including T cells, B cells, natural killer cells, and monocytes. It is approved for the treatment of active relapsing-remitting multiple sclerosis (RRMS) and has demonstrated superior efficacy compared to interferon beta-1a in reducing relapse rates in RRMS patients [1,2]. Despite its therapeutic benefits, alemtuzumab is associated with significant adverse effects, including infusion reactions, infections, and autoimmune disorders [3,4]. Thyroid dysfunction is one of the most common autoimmune complications, occurring in approximately 20% to 30% of treated patients, with Graves’ disease being the most frequent manifestation [5]. Thyrotoxicosis refers to the clinical state of excess circulating thyroid hormones, whereas thyroid storm represents its most severe life-threatening form, characterized by multiorgan dysfunction and decompensated hypermetabolism. However, thyroid storm in the context of alemtuzumab therapy is extremely rare, with only one other case reported to date [6].

This case is distinct because it describes an alemtuzumab-induced Graves’ thyrotoxicosis that progressed to a treatment-resistant thyroid storm, ultimately requiring surgical intervention, and revealed an incidental papillary thyroid carcinoma on histopathology. Highlighting this combination underscores the need for awareness of severe refractory autoimmune thyroid events and the potential coexistence of malignancy following alemtuzumab therapy. Recent evidence also suggests that alemtuzumab therapy may increase the incidence of thyroid-related malignancies, particularly papillary thyroid carcinoma [7]. Notably, these cancers are often detected incidentally during histopathological examination following thyroidectomy for autoimmune thyroid disease.

## Case presentation

A 38-year-old woman with a history of RRMS treated with alemtuzumab presented acutely with palpitations, chest tightness, dizziness, and light-headedness. On examination, she was clinically symptomatic with marked tachycardia (heart rate 160/min). Laboratory investigations revealed severe thyrotoxicosis (free T4 >100 pmol/L (reference: 12-22 pmol/L), TSH <0.01 mU/L (reference: 0.4-4.0 mU/L)). Troponin was elevated at 71 ng/L (reference: <14 ng/L), attributed to cardiac strain from tachycardia. Electrocardiography confirmed sinus tachycardia without ischemic changes.

Initial management focused on controlling hyperthyroidism and adrenergic symptoms. Her carbimazole dose was increased to 30 mg three times daily, but there was no clinical or biochemical improvement, with persistent severe thyrotoxicosis (free T4 >100 pmol/L) and ongoing tachycardia. Propranolol was commenced and titrated up to 80 mg four times daily for rate control, yet her heart rate remained above 130 bpm. The patient developed features consistent with thyroid storm, prompting the addition of intravenous corticosteroids and Lugol’s iodine (0.2 mL three times daily) to inhibit thyroid hormone synthesis and release.

Despite maximal medical therapy, the patient remained highly symptomatic and met criteria for resistant thyroid storm. A structured assessment using the Burch-Wartofsky Point Scale (Table 1) supported the clinical suspicion, with a total score indicative of high probability [8]. Serial T4 measurements demonstrated persistently elevated levels despite aggressive therapy, eventually trending toward normal following preoperative preparation (see Table 2). An echocardiogram excluded structural cardiac disease or regional wall motion abnormalities. Given the severity and refractoriness of her condition, urgent surgical intervention was planned. Preoperative optimization was achieved with Lugol’s iodine, and euthyroidism was established (free T4: 22 pmol/L, TSH <0.01 mU/L) within 20 days of presentation. She subsequently underwent total thyroidectomy without complication. Postoperatively, her calcium levels remained stable, and she was initiated on levothyroxine (100 µg daily).

**Table 1:** Burch–Wartofsky Point Scale Assessment Including Reference Criteria and Patient-Specific Values

Parameter	Reference Criteria	Patient Value	Score Assigned
Thermoregulatory dysfunction	<37.8°C = 5	37.8°C	10
	37.8–38.3°C = 10		
	38.4–38.8°C = 15		
	38.9–39.4°C = 20		
	39.4–39.9°C = 25		
	≥40°C = 30		
Central nervous system effects	Mild = 10	Present (mild)	10
	Moderate = 20		
	Severe = 30		
Gastrointestinal-hepatic dysfunction	Moderate (delirium, psychosis, extreme lethargy) = 10	Present (moderate)	10
	Severe (jaundice) = 20		
Cardiovascular dysfunction (HR)	90–109 = 5	160 bpm	25
	110–119 = 10		
	120–129 = 15		
	130–139 = 20		
	≥140 = 25		
Congestive heart failure (CHF)	Mild = 5	Absent	0
	Moderate = 10		
	Severe = 15		
Atrial fibrillation	Present = 10	Absent	0
	Absent = 0		
Precipitating event	Present = 10	Alemtuzumab	10
	Absent = 0		
Total Score			65

**Table 2:** Serial Free T4 Measurements From Day of Admission in a Patient With Thyroid Storm This table presents the patient’s serum free thyroxine (T4) levels measured at serial time points following hospital admission. Values are expressed in picomoles per liter (pmol/L). Reference range: 12–22 pmol/L. T4 = thyroxine; pmol/L = picomoles per liter.

Test	Day 0	Day 7	Day 11	Day 15	Day 18	Day 20	Reference values
T4 (pmol/L)	>100	91	55	36	29	22	12–22 pmol/L

Histopathological examination of the resected thyroid confirmed Graves' disease, underscoring the recognized association between alemtuzumab therapy and the development of autoimmune thyroid dysfunction. Additionally, an incidental papillary thyroid carcinoma (pT1a Nx Vo Mx Ro) was identified. The regional thyroid cancer multidisciplinary team recommended conservative management for the carcinoma given its incidental discovery and favorable pathological features.

## Discussion

This case highlights a rare but severe complication of alemtuzumab therapy: thyroid storm secondary to Graves' disease [9,10]. Thyroid dysfunction is a well-recognized adverse effect of alemtuzumab due to immune reconstitution following lymphocyte depletion [11]. Graves' disease accounts for up to 70% of thyroid dysfunction cases associated with alemtuzumab therapy. However, thyroid storm, which is a medical emergency characterized by decompensated thyrotoxicosis, has only been reported once before in this context.

**Figure 1:**
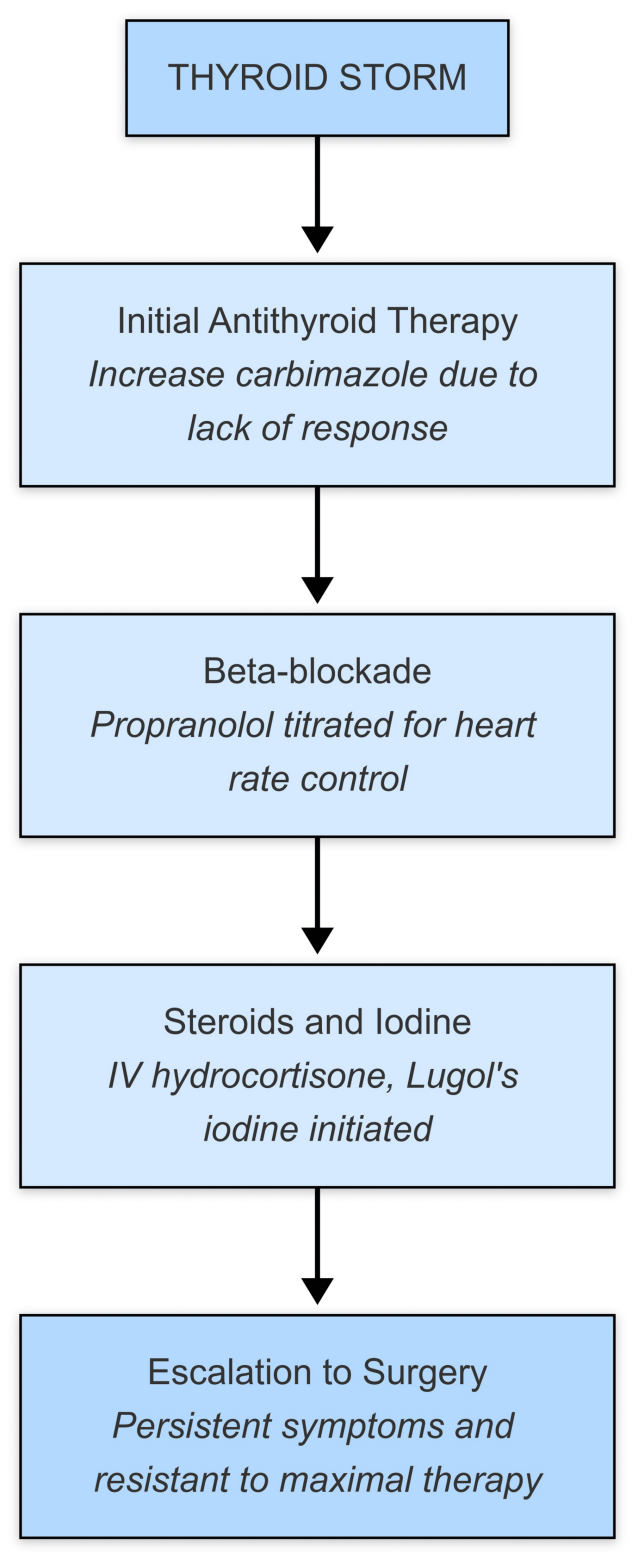
Stepwise Management of Thyroid Storm in a Patient With Alemtuzumab-Induced Thyrotoxicosis Illustrates Escalation From Medical to Surgical Intervention Following Failure of Standard Therapies

The pathophysiology of alemtuzumab-induced thyroid dysfunction involves immune reconstitution syndrome (IRS), which triggers autoimmunity against thyroid antigens. In this patient, early recognition and aggressive management were critical to preventing life-threatening complications. Despite escalating doses of antithyroid medications and adjunct therapies such as Lugol's iodine and corticosteroids, definitive surgical intervention was required due to refractory thyrotoxicosis (Figure 1).

Histopathology revealed an incidental papillary thyroid carcinoma (PTC), raising important considerations about the potential association between alemtuzumab therapy and thyroid malignancy [12-14]. Although reports of PTC following alemtuzumab are rare and an overall increase in malignancy rates has not been confirmed, chronic autoimmune stimulation and thyroid inflammation may contribute to carcinogenesis in predisposed individuals [15]. Current guidelines recommend evaluation of thyroid nodules with ultrasound and fine-needle aspiration when indicated to facilitate early detection [16]. In this context, clinicians should remain vigilant for both autoimmune and neoplastic thyroid complications following alemtuzumab therapy [17].

In summary, this case demonstrates that alemtuzumab-induced Graves' thyrotoxicosis can progress to a treatment-resistant thyroid storm requiring surgery. Key clinical lessons include close endocrine monitoring after alemtuzumab, early recognition of rapid thyrotoxicosis progression, and multidisciplinary management to optimize outcomes.

## Conclusions

This case highlights the critical importance of vigilant thyroid function monitoring in patients receiving alemtuzumab for RRMS. Routine TFTs should be performed before treatment and every three months for at least four years after initiation to ensure early detection of dysfunction. In alemtuzumab-induced Graves' disease, rapid fluctuations between hyperthyroidism and hypothyroidism necessitate frequent TFT assessments (e.g., monthly for six months after starting levothyroxine). Early intervention with antithyroid drugs or hormone replacement is essential, although some patients may develop resistance to medical therapy and progress to severe thyroid storm requiring surgical management.

From this case, three key clinical lessons emerge: first, severe autoimmune thyroid disease can follow alemtuzumab therapy and may be treatment resistant; second, timely surgical intervention can be lifesaving when conventional therapy fails; and third, ongoing thyroid surveillance and patient education on symptom awareness are vital preventive measures. Thyroid storm in this setting requires aggressive medical stabilization followed by definitive thyroidectomy in refractory cases. Finally, effective multidisciplinary coordination among endocrinologists, neurologists, anesthetists, and surgeons is essential to ensure safe and timely management and to improve outcomes for patients with complex alemtuzumab-related thyroid disease.

## References

[1] Coles AJ, Twyman CL, Arnold DL (2012). Alemtuzumab for patients with relapsing multiple sclerosis after disease-modifying therapy: a randomised controlled phase 3 trial. Lancet.

[2] Havrdova E, Horakova D, Kovarova I (2015). Alemtuzumab in the treatment of multiple sclerosis: key clinical trial results and considerations for use. Ther Adv Neurol Disord.

[3] Willis M, Robertson NP (2014). Drug safety evaluation of alemtuzumab for multiple sclerosis. Expert Opin Drug Saf.

[4] Guarnera C, Bramanti P, Mazzon E (2017). Alemtuzumab: a review of efficacy and risks in the treatment of relapsing remitting multiple sclerosis. Ther Clin Risk Manag.

[5] Mahzari M, Arnaout A, Freedman MS (2015). Alemtuzumab induced thyroid disease in multiple sclerosis: a review and approach to management. Can J Neurol Sci.

[6] AlShehri S, Alajmi S, Ekhzaimy A, Aldawas S, Alalwan M (2022). Thyroid storm in a patient with alemtuzumab-induced Graves’ disease: a case report. Cureus.

[7] Ibitoye R, Wilkins A (2014). Thyroid papillary carcinoma after alemtuzumab therapy for MS. J Neurol.

[8] Burch HB, Wartofsky L (1993). Life-threatening thyrotoxicosis. Thyroid storm. Endocrinol Metab Clin North Am.

[9] Ueland GÅ, Ueland HO, Stokland AM (2024). Prevalence, risk factors, and clinical and biochemical characteristics of alemtuzumab-induced Graves disease. J Clin Endocrinol Metab.

[10] Manso J, Muller I, Mian C (2025). Clinical management of alemtuzumab-induced autoimmune thyroid diseases: a narrative review. Eur Thyroid J.

[11] Rolla S, Maglione A, De Mercanti SF, Clerico M (2020). The meaning of immune reconstitution after alemtuzumab therapy in multiple sclerosis. Cells.

[12] Steingo B, Al Malik Y, Bass AD (2020). Long-term efficacy and safety of alemtuzumab in patients with RRMS: 12-year follow-up of CAMMS223. J Neurol.

[13] Frau J, Coghe G, Lorefice L, Fenu G, Musu L, Cocco E (2019). Efficacy and safety of alemtuzumab in a real-life cohort of patients with multiple sclerosis. J Neurol.

[14] Zmira O, Halpern AI, Abraham L, Achiron A (2021). Efficacy and safety of alemtuzumab treatment in a real-world cohort of patients with multiple sclerosis. Acta Neurol Belg.

[15] Ziemssen T, Thomas K (2017). Alemtuzumab in the long-term treatment of relapsing-remitting multiple sclerosis: an update on the clinical trial evidence and data from the real world. Ther Adv Neurol Disord.

[16] Coles AJ, Jones JL, Vermersch P (2022). Autoimmunity and long-term safety and efficacy of alemtuzumab for multiple sclerosis: benefit/risk following review of trial and post-marketing data. Mult Scler.

[17] Decallonne B, Bartholomé E, Delvaux V (2018). Thyroid disorders in alemtuzumab-treated multiple sclerosis patients: a Belgian consensus on diagnosis and management. Acta Neurol Belg.

